# A Unique Case of Tension Empyema Caused by Prevotella denticola

**DOI:** 10.7759/cureus.25853

**Published:** 2022-06-11

**Authors:** Masi Javeed, Fahd Rahmouni Idrissi, Nek Nazary

**Affiliations:** 1 Internal Medicine, HCA Florida Bayonet Point Hospital, Hudson, USA; 2 Surgery, HCA Florida Bayonet Point Hospital, Hudson, USA; 3 Family Medicine, HCA Florida Bayonet Point Hospital, Hudson, USA

**Keywords:** thoracostomy tube, tension, pleural empyema, thoracic empyema, prevotella

## Abstract

A 45-year-old male presented to the emergency department after being found unresponsive. Vitals, laboratory findings, and chest X-ray revealed concern for tension empyema. Thoracostomy was performed, and hemodynamics subsequently improved. Later, *Prevotella denticola* was cultured. This is the first known reported case of tension *Prevotella denticola* empyema.

## Introduction

Empyema is a collection of pus in the pleural space. If there is acute accumulation of enough pus, it can cause severe respiratory distress, mediastinal shift, and hemodynamic compromise [1]. While empyema is a common occurrence, it rarely causes tension physiology [1]. One of the earliest known cases was reported in 1986, and yet there are still fewer than 10 reported cases of tension empyema across the published literature [1-8]. A few studies revealed streptococcus and staphylococcus cultured from the empyema [5,7]. However, we present the first case of tension *Prevotella denticola* empyema.

## Case presentation

**Figure 1:**
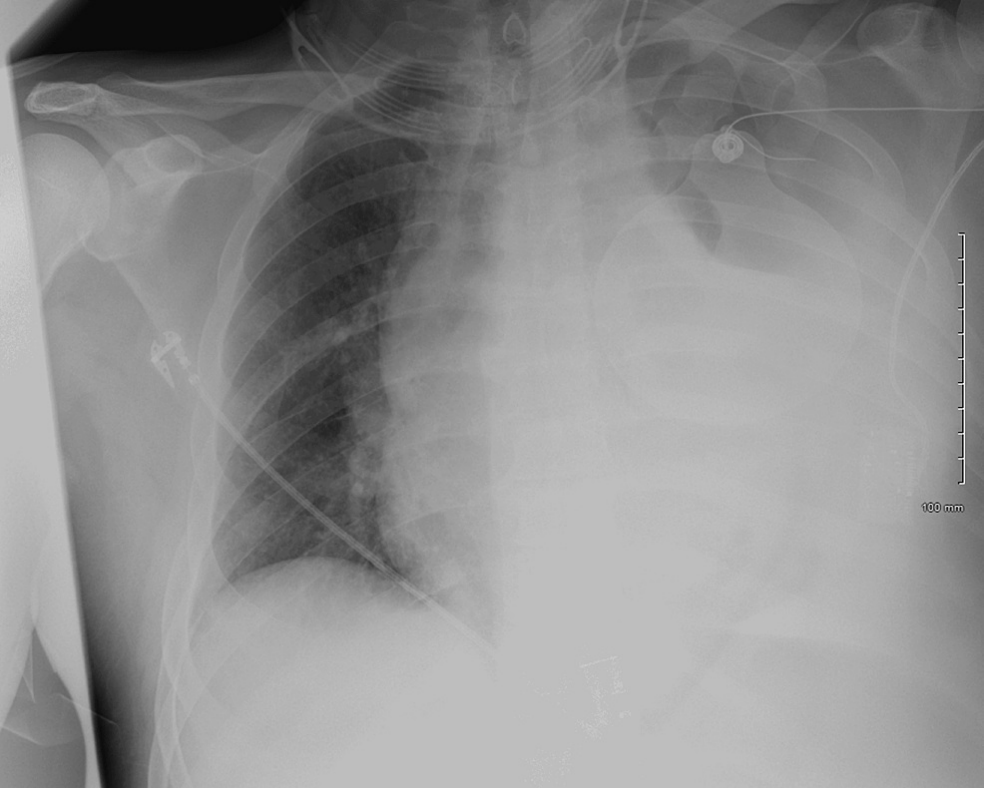
Initial chest X-ray revealed a large left-sided pleural effusion with significant rightward tracheal deviation and a diminished right lung field.

A 45-year-old Caucasian male presented to the emergency department by ambulance after being found unresponsive. Preceding events as well as medical history were not known. No family members or friends could be identified. Significant vitals included a temperature of 39.1 degrees Celsius, respiratory rate of 49 breaths per minute, oxygen saturation of 71%, blood pressure of 79/52 millimeters of mercury, and heart rate of 126 beats per minute. Physical examination revealed diminished left-sided breath sounds, left-sided dullness to percussion, and asymmetrical chest expansion. Significant laboratory findings included an elevated neutrophilic white blood cell count of 55,200 per microliter, elevated lactic acid of 5.7 millimoles per liter, and an elevated C-reactive protein of 22 milligrams per deciliter. Chest X-ray revealed a large left-sided effusion as well as rightward tracheal deviation (Figure 1).

**Figure 2:**
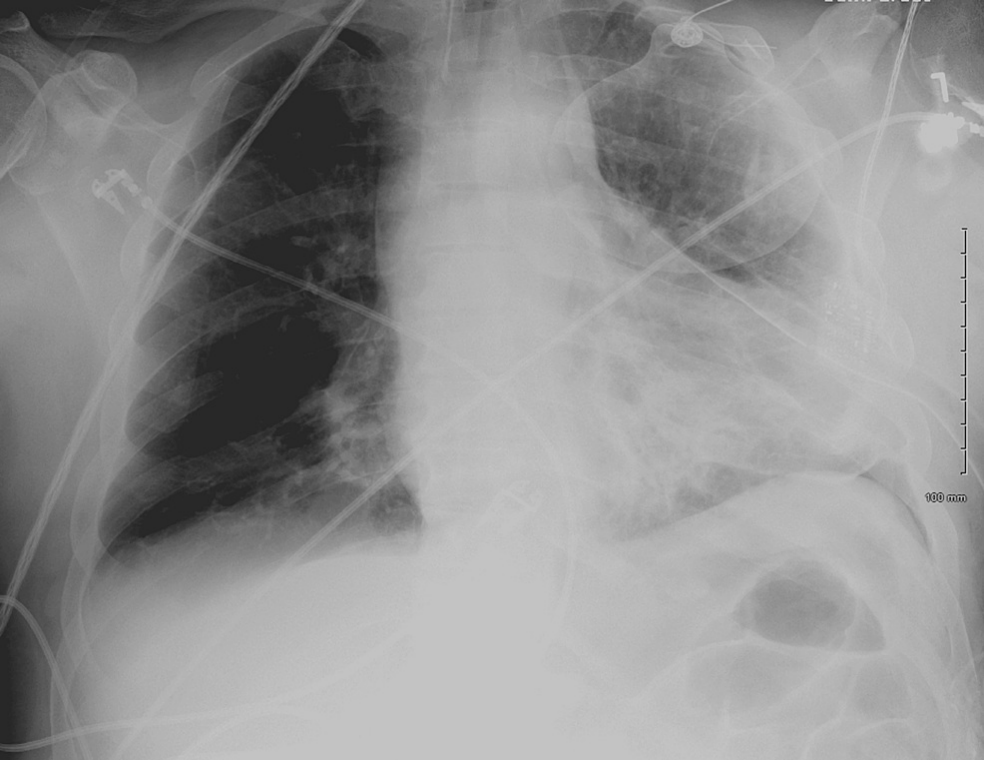
Subsequent chest X-ray after thoracostomy revealed a decreased left-sided pleural effusion, a decreased tracheal deviation, and an increased right lung field.

The patient was found to have a Glasgow coma scale of 5; specifically he had the following scores: eyes 2, verbal 2, and motor 1. The patient then underwent rapid sequence intubation and was placed on mechanical ventilation. In addition, a chest tube was inserted in the fifth intercostal space along the mid-axillary line. Over 3 liters of purulent foul-smelling fluid was obtained. Hemodynamics improved subsequently, and repeat chest X-ray showed improvement as well (Figure 2).

The patient was bolused 3 liters of normal saline over 90 minutes and maintained on intravenous fluids as well as started on vancomycin and piperacillin/tazobactam prior to being admitted. The patient was later found to have abundant *Prevotella denticola* on cultures.

## Discussion

Tension empyema may occur as a complication of pneumonia or a lung abscess [1]. *Streptococcus* is the most common causative bacteria [2]. A large volume of pus can cause an inflammatory and fibrotic response that entraps the lung and shifts the mediastinal organs including the trachea. It can also increase intrathoracic pressure, which, in turn, can reduce venous return and cardiac output.

Older age, male sex, chronic obstructive pulmonary disease, diabetes, alcoholism, and an immune-compromised state are potential risk factors [3]. Symptoms may include chest pain, cough, and severe dyspnea. Vitals may reveal fever, tachycardia, tachypnea, and hypotension. Physical examination findings may include absent breath sounds, a dull percussion note, and tracheal deviation to the opposite hemithorax [1]. Chest X-ray and ultrasound may be helpful in revealing a large collection of fluid. However, diagnosis and immediate treatment is through urgent thoracostomy and culturing of the resulting fluid [1]. Antibiotics may include cephalosporins, antipseudomonal penicillins, carbapenems, and metronidazole [7].

## Conclusions

Tension empyema is a very rare, life-threatening condition. This is the only reported case where *Prevotella denticola* was the causative bacteria. Urgent thoracostomy should be performed to both diagnose and treat this condition. Appropriate antibiotics should be administered as well.
